# Incidence of bronchiectasis in patients with acromegaly: a cohort study

**DOI:** 10.3389/fendo.2024.1362950

**Published:** 2024-08-16

**Authors:** Hyun-Il Gil, Kyungdo Han, Sangmo Hong, Cheol-Young Park

**Affiliations:** ^1^ Division of Pulmonary and Critical Care Medicine, Department of Internal Medicine, Kangbuk Samsung Hospital, Sungkyunkwan University School of Medicine, Seoul, Republic of Korea; ^2^ Department of Statistics and Actuarial Science, Soongsil University, Seoul, Republic of Korea; ^3^ Department of Internal Medicine, Guri Hospital, College of Medicine, Hanyang University, Guri-si, Republic of Korea; ^4^ Division of Endocrinology and Metabolism, Department of Internal Medicine, Kangbuk Samsung Hospital, Sungkyunkwan University School of Medicine, Seoul, Republic of Korea

**Keywords:** non-cystic fibrosis bronchiectasis, acromegaly, small airway disease, growth hormone, cohort study

## Abstract

**Objective:**

Associations between acromegaly and several respiratory diseases, such as obstructive lung disease or sleep apnea, have been suggested, but the relationship between bronchiectasis and acromegaly is unclear. We investigated whether acromegaly is related to the development of bronchiectasis.

**Materials and methods:**

Using the Korean National Health Insurance System database between 2006 and 2016, we studied the relationship between acromegaly and bronchiectasis in patients with acromegaly (n=2593) and controls (1:5 age- and sex-matched subjects without acromegaly, n=12965) with a mean follow-up period of 8.9 years. Cox proportional hazards regression analysis was used to assess the risk of bronchiectasis in patients with acromegaly compared with controls after adjusting for age, sex, household income, place, type 2 diabetes, hypertension, and dyslipidemia.

**Results:**

The mean age of the participants was 47.65 years, and male subjects comprised 45.62% of the cohort. The incidence rate of bronchiectasis in patients with acromegaly was 3.64 per 1,000 person-years and was significantly higher than that in controls (2.47 per 1,000 person-years) (log-rank test p = 0.002). In multivariable Cox proportional hazards regression modeling, the risk of bronchiectasis was significantly higher in patients with acromegaly than that in controls (HR: 1.49; 95% CI: 1.15–1.94, p = 0.0025) after adjusting for age, sex, household income, place, type 2 diabetes, hypertension, and dyslipidemia.

**Conclusions:**

Our results suggest that acromegaly may be associated with bronchiectasis.

## 1 Introduction

Non-cystic fibrosis bronchiectasis is a chronic lung disease that causes damage and widening of the airways, leading to cough, difficulty breathing, production of excess airway secretion, and increased risk of lung infections (1). Over the past decades, the increased availability of chest imaging has led to increased awareness of bronchiectasis, and improvements in specific treatments to reduce the complications and progression of bronchiectasis have led to increased medical interest in bronchiectasis, which has long been an orphan disease (2, 3). Various etiologies of bronchiectasis have been recognized, including chronic lung infections, small airway diseases, genetic conditions, and immune system disorders, but an etiology is identified only in approximately 60% of bronchiectasis patients (4).

Acromegaly is a rare hormonal disorder caused by excessive secretion of growth hormone (GH) and insulin-like growth factor-1 (IGF-1), which usually occurs in benign pituitary tumors (5).. This condition causes an overgrowth of bones and tissues, which leads to a variety of physical symptoms, including enlargement of the hands, feet, and facial features, as well as other metabolic problems, such as diabetes and cardiovascular disease, which are associated with significant morbidity and mortality (6, 7).

There is no known large-scale, longitudinal study on the relationships between certain pulmonary diseases and acromegaly. As one of comorbidities of acromegaly, bronchiectasis was first reported in patients with acromegaly by Nabarro et al. as early as 1987 (8), but never investigated any further until the primarily radiologically driven work in 2013 (9). This study reported that 35% of patients with acromegaly had bronchiectasis (9), and the prevalence of bronchiectasis was much higher than the reported overall prevalence of bronchiectasis in the general population (approximately 0.1% to 0.5%) (10–12). However, these findings were difficult to generalize because the study was small with a cross-sectional design. Some studies suggest that patients with acromegaly may be at higher risk for respiratory problems, such as sleep apnea and small airway diseases (13–15). Sleep apnea or small airway disease may increase the risk of bronchiectasis over time (16, 17), however, this relationship is not fully understood. Although there are many findings showing that acromegaly is associated with increased lung volume, decreased lung compliance, and airway obstruction, there is still insufficient evidence to link these lung conditions to bronchiectasis (18, 19). As mentioned above, there are several findings suggesting the possibility of bronchiectasis occurring as a comorbidity of acromegaly, but research on this topic is still lacking. In this longitudinal study, we examined the incidence of bronchiectasis in patients with acromegaly using a large National Health Information Database (NHID).

## 2 Materials and methods

### 2.1 Study database

Our data were extracted from the NHID of the Korean National Health Insurance Service (NHIS), a single-payer universal healthcare system of Republic of Korea that covers almost all Korean citizens. This database is also linked to other healthcare programs operated by the Korean government, such as National Health Screening Program, and the Rare Incurable Disease Registry (20).

Since 2009, the government of Republic of Korea has operated a rare incurable disease registry. A ‘rare incurable’ disease refers to that with very low or unknown incidence and that is difficult to diagnose or treat. A disease must meet strict criteria to be included in this registry. Only diseases that meet the conditions below, including radiological studies, biochemistry, immunology, microbiology, histology, and clinical diagnosis by a clinician, can be registered (21).. In this study, we selected patients with acromegaly from this rare incurable disease registry.

The study protocol was approved by the Institutional Review Board of Kangbuk Samsung Hospital (KBSMC 2022-07-033). Informed consent was waived due to the observational nature of the study and anonymization and de-identification of patient information prior to analysis.

### 2.2 Definitions of acromegaly, bronchiectasis, and comorbidities

In this study, acromegaly was defined as a case in a patient with a history of outpatient treatment or hospitalization according to the International Classification of Diseases, 10th Revision (ICD-10) diagnosis code (E22.0 for acromegaly) and the code of the rare incurable disease registry (V112 for acromegaly). For acromegaly, a compatible radiologic finding (computed tomography or magnetic resonance imaging scan) and a biochemical test with a glucose tolerance test are both required for a patient to receive these disease codes.

Bronchiectasis was defined by the following criteria: (1) age ≥ 20 years (to rule out childhood bronchiectasis due to other congenital diseases that may affect the lungs); (2) at least one claim under ICD-10 code J47 for bronchiectasis; and (3) exclusion of patients with cystic fibrosis (ICD-10 code E84) or congenital bronchiectasis (ICD-10 code Q334) (10).

Metabolic comorbidities associated with acromegaly were defined using the NHID. The presence of type 2 diabetes mellitus was defined based on claims for ICD-10 codes (E11–14) and by prescription of antidiabetic medication. The presence of hypertension was defined based on claims for ICD-10 codes (I10, I11, I12, I13, or I15) and by prescription of antihypertensive medication, and the presence of dyslipidemia was defined based on claims for ICD-10 code (E78) and by prescription of lipid-lowering agents.

### 2.3 Study subjects

In this nationwide, observational, retrospective cohort, we initially screened 2857 subjects with acromegaly from 2006 to 2016. Of these, a total of 264 patients were excluded from our study, including 148 patients under 20 years of age, 65 patients with cystic fibrosis or congenital bronchiectasis, and 51 patients with missing complete data. Finally, 2593 acromegaly patients who met the study criteria were included in the acromegaly group. A 1:5 age-matched and sex-matched control cohort without acromegaly was randomly assigned, and 12965 subjects were enrolled in the control group.

### 2.4 Data analysis

Baseline characteristics were analyzed using descriptive statistics. Categorical variables were described as frequencies and percentages. Continuous variables were described as means (± standard deviations) for normally distributed data. We compared baseline characteristics of patients with acromegaly at diagnosis and in 1:5 age- and sex-matched controls without acromegaly at enrollment. Continuous variables were compared using the independent sample t-test, while categorical variables were compared using the χ2 test. The follow-up duration of each group was obtained. The incidence rates of bronchiectasis were estimated for each group over the entire follow-up period. Incidence curves were estimated using the Kaplan–Meier method, and the log-rank test was also performed. The risks of bronchiectasis were analyzed using Cox proportional hazards regression analysis while controlling for baseline covariates. If the *p* value was less than 0.05, the null hypothesis was rejected. Analyses were performed using SAS 9.4 (SAS Institute, Cary, NC, USA) and R, version 3.4.1 (The R Foundation for Statistical Computing, Vienna, Austria, R-project.org).

## 3 Results

### 3.1 Baseline characteristics of subjects

**Table 1** shows the baseline characteristics of the 2593 patients with acromegaly at the time of diagnosis and the baseline characteristics of 12965 1:5 age- and sex-matched controls at the time of matching. The mean age of the subjects was 47.65 ± 13.83 years, and 45.6% were male. Patients with acromegaly had a higher prevalence of diabetes (28.27% vs. 5.98%, p < 0.0001), hypertension (38.60% vs. 17.48%, p <0.0001), and dyslipidemia (17.08% vs. 9.09%, p <0.0001) compared with age- and sex-matched controls.

**Table 1:** Baseline demographics and metabolic comorbidities of patients with acromegaly and age- and sex-matched controls without acromegaly.

	Controls (n = 12965)	Acromegaly (n = 2593)	*p* value
Age: years	47.65 ± 13.83	47.65 ± 13.83	1
Sex: male	5915 (45.62)	1183 (45.62)	1
Household income: low 25%	2761 (21.3)	577 (22.25)	0.2788
Place: urban	6159 (47.5)	1230 (47.44)	0.9485
Type 2 diabetes mellitus	775 (5.98)	733 (28.27)	<0.0001
Hypertension	2266 (17.48)	1001 (38.6)	<0.0001
Dyslipidemia	1178 (9.09)	443 (17.08)	<0.0001
Follow-up duration: years	8.92 ± 3.45	8.70 ± 3.55	0.0030

Values are given as mean ± standard deviation or number (%).

P-value based on the t test for continuous variables and the χ^2^ test for categorical variable.

### 3.2 Acromegaly and bronchiectasis

**Figure 1:**
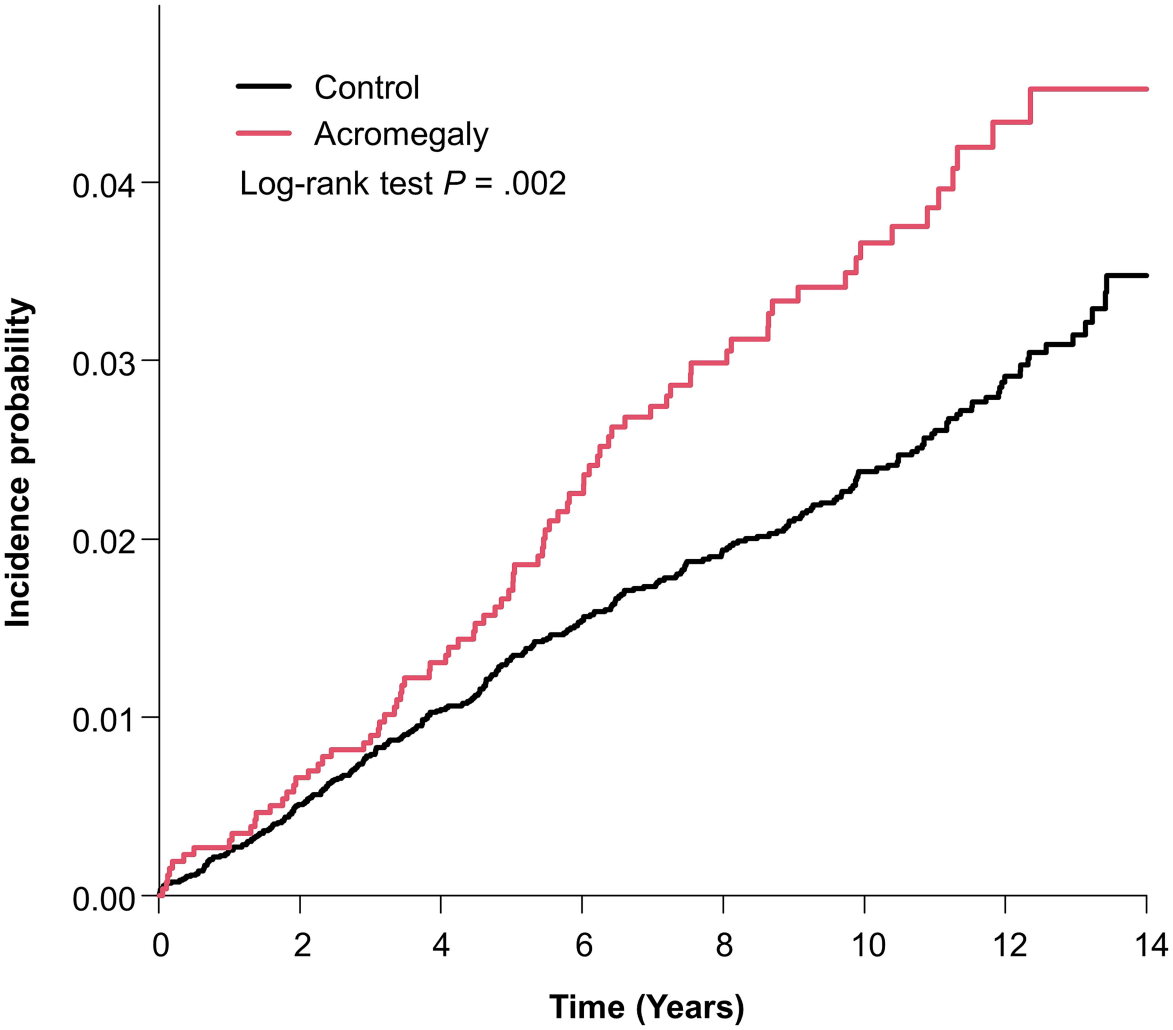
Kaplan–Meier curve of incidence probability of bronchiectasis in patients with acromegaly and controls.

**Table 2** shows the cumulative prevalence, incidence rate, and hazard ratio (HR) of bronchiectasis in the acromegaly group and control group. The bronchiectasis was identified in 82 (3.16%) patients with acromegaly and 286 (2.21%) controls during a mean follow-up of 8.70 ± 3.55 years and 8.92 ± 3.45 years, respectively. The incidence rate of bronchiectasis was 3.64 per 1000 person-years in patients with acromegaly and 2.47 per 1000 person-years in controls. The incidence rate of bronchiectasis in patients with acromegaly was significantly higher than in controls (log-rank test: p = 0.002). **Figure 1** shows the results of Kaplan–Meier survival analysis for the cumulative incidence of bronchiectasis.

**Table 2:** Frequency, incidence rate, and hazard ratio of bronchiectasis in patients with acromegaly and controls. .

	No. patients	BE	Duration (person-year)	Incidence rate (per 1,000 person-year)	Hazard ratio (95% confidence interval)			
					P value	Model 1	P value	Model 2
Controls	12965	286	115629	2.47		1 (ref.)		1 (ref.)
Acromegaly	2593	82	22553	3.64	0.0010	1.51 (1.18–1.93)	0.0025	1.49 (1.15–1.94)

BE, Bronchiectasis.

Model 1: Adjusted for age and sex.

Model 2: Adjusted for age, sex, household income, place, type 2 diabetes, hypertension, and dyslipidemia.

In multivariable Cox proportional hazards regression, the risk of bronchiectasis was significantly higher in acromegaly group than that in the control group (HR: 1.51; 95% CI: 1.18–1.93, p = 0.001) after adjusting for age and sex (model 1) and also higher (HR: 1.49; 95% CI: 1.15–1.94, p = 0.0025) after adjusting for age, sex, household income, place, type 2 diabetes, hypertension, and dyslipidemia (model 2).

Subgroup analysis was performed to determine the hazard ratio of bronchiectasis and the presence of interactions between variables based on the subjects’ demographic characteristics (age, gender, household income, and location) and metabolic comorbidities (type 2 diabetes, hypertension, dyslipidemia) (**Supplementary Table 1**). In subgroup analysis, the interaction between variables was not statistically significant. The HR of bronchiectasis was significantly higher in the acromegaly group, regardless of age, sex, income, place, type 2 diabetes, hypertension, and dyslipidemia.

## 4 Discussion

In this nationwide, retrospective cohort study, we investigated the association between acromegaly and bronchiectasis. During the follow-up period of approximately 8.9 years, the risk of bronchiectasis was higher by 1.49-fold (95% confidence interval (CI): 1.15–1.94) in patients with acromegaly than in controls, and this result was significant after adjusting confounding variables (age, sex, household incomes, place, type 2 diabetes, hypertension, and dyslipidemia). While the interpretation of findings of the retrospective study requires caution, the results of our study, which included a large number of acromegaly patients (n=2593), suggest that acromegaly may be associated with the development of bronchiectasis.

The prevalence of bronchiectasis in Asian countries is reported to be higher than in Western countries, which is thought to be due to the higher prevalence of tuberculosis (10).. In the large-scale population-based cohort study of the incidence of bronchiectasis in Korea by Choi et al., the annual incidence of bronchiectasis in Korea from 2005 to 2013 ranged from 1.47 to 2.29 cases per 1000 person (22), which was significantly higher than 0.21 in the United States (23). In our study, which analyzed data from 2006 to 2016 in Korea, the incidence in the control group was similar to the previous study, with an incidence of 2.47 people per 1000 person-years. In the acromegaly patient group, the incidence of bronchiectasis was significantly higher with 3.64 patients per 1000 person-years. Therefore, even considering that the study was conducted on patients in a country with a high prevalence of tuberculosis, bronchiectasis occurred more frequently in patients with acromegaly.

Large retrospective studies of acromegaly patients indicate an average 10-year reduction in life expectancy due to cardiovascular, cerebrovascular, metabolic, and respiratory comorbidities (24). Among respiratory comorbidities, sleep apnea has been identified as the main cause of respiratory complications and death in patients with acromegaly (25). However, acromegaly causes not only sleep apnea, but also reduced lung function and structural lung damage. In a study conducted by Camilo et al., bronchiectasis was observed in approximately 35% of patients with acromegaly, which was the second most common abnormality found on chest computed tomography (CT) after air-trapping. However, the small number of cases and the cross-sectional nature of the study made it difficult to generalize the results (9). Also, because this study used high-resolution computed tomography and was performed by expert radiologists, the reporting of these results may be subject to attentional bias. If the extent of bronchiectasis found was small, it may not be clinically significant. In another study analyzing changes in lung function and structure in patients with acromegaly, chest CT showed no changes in lung parenchyma. However, in patients with acromegaly, the ability to diffuse carbon monoxide was found to be reduced (26). The researchers suggested alveolar membrane damage or microvascular damage as a mechanism to explain the results of this study that showed only functional decline and no change in lung parenchyma, which are contradictory to previous studies. As the results of previous studies on structural lung complications in patients with acromegaly were inconsistent and the mechanisms were unclear, we conducted this study to determine whether structural lung damage occurs in patients with acromegaly in the real world. While the frequency of bronchiectasis was lower in our study than the previous one, our large-scale, longitudinal study involving more than 2000 patients with this rare disease showed that the incidence of bronchiectasis was higher than that in the general population.

Common metabolic complications of acromegaly such as hypertension, diabetes, and dyslipidemia are also common in bronchiectasis. In a systematic review including 40 studies on comorbidities of bronchiectasis, cardiovascular disease (33%), hypertension (30%), and diabetes (28%) were common in bronchiectasis patients (27). Some studies have suggested that people with diabetes may have an increased risk of developing respiratory infections and chronic lung diseases such as bronchiectasis (28, 29), and dysfunction of regulatory proteins involved in cholesterol metabolism or diet-induced dyslipidemia may contribute to the pathogenesis of acute lung injury, asthma, pneumonia, and other lung disorders (30). However, there is no known distinct causal relationship between the metabolic complications of acromegaly and bronchiectasis. In the Cox proportional hazards regression analysis results of our study, there was almost no change in HR of bronchiectasis before (HR: 1.51; 95% CI: 1.18–1.93, Model 1) and after (HR: 1.49, 95% CI: 1.15–1.94, Model 2) adjusting for diabetes, hypertension, and dyslipidemia. This suggests that these metabolic comorbidities may have little association with the development of bronchiectasis. The pathogenesis between bronchiectasis and acromegaly seems to involve a different mechanism from that of the metabolic complications of acromegaly.

In acromegaly, excessive secretion of GH stimulates hepatic IGF-1 production, and IGF-1 affects various tissues to result in various phenotypes of acromegaly. While the role of IGF-1 signaling and clinical relevance of serum IGF-1 level in lung disease have been studied, there are many unknowns (31). In some congenital or genetic lung diseases such as bronchopulmonary dysplasia or cystic fibrosis, serum IGF-1 level was decreased in patients compared with the healthy population (32–35). In contrast to these diseases, upregulated IGF-1 expression or signaling is observed in many fibrotic lung diseases such as idiopathic pulmonary fibrosis, late-stage sarcoidosis, and secondary fibrosis (31, 36–40). However, there is no direct evidence for a role of IGF-1 in non-cystic fibrosis bronchiectasis. Few studies have investigated IGF-1 status in patients with acromegaly and bronchiectasis. In a study by Rodrigues et al. involving 36 acromegaly patients and 24 control subjects, which investigated the relationship between acromegaly and structural lung abnormalities, there was no statistical difference between serum IGF-1 level in patients with or without bronchiectasis (41). However, the study was cross-sectional, the number of cases in the study was too small, and the frequency of bronchiectasis was much higher than that of the general population. Unfortunately, we could not analyze the serum IGF-1 level in our study. Due to the nature of the claims data, we were unable to analyze how much higher IGF-1 levels were in the acromegaly group compared to the control group. However, the glucose tolerance test result was suitable to determine acromegaly for the patient to be included in the rare incurable disease registry, so the patients included in this study were those with high serum IGF-1 level. Therefore, although the role of IGF-1 cannot be proven in situations where exact values ​​of IGF-1 levels are not available, it can be hypothesized that high IGF-1 levels may be qualitatively, if not quantitatively, involved in the development of bronchiectasis. In other words, this study suggests the possibility that there is a link between the two diseases. Although it is difficult to design large-scale prospective studies for rare diseases such as acromegaly, further studies will be needed to demonstrate the role of IGF-1 in the pathogenesis of acromegaly-related bronchiectasis.

The advantages of our study are that this study was longitudinal and included a large number of subjects with the very rare disease acromegaly (n = 2593) and age- and sex-matched controls (n = 12965). In addition, available confounding factors were adjusted for analysis, and subgroup analysis was also performed. However, this study has several limitations. First, the study design is retrospective and observational. Due to the limitations of the study design, there is a possibility that there are unidentified confounding factors. Nevertheless, we tried to include variables that can be analyzed as much as possible in large-scale claims data targeting rare diseases for which large-scale prospective studies are impossible. Second, because we defined acromegaly, bronchiectasis, and comorbidities using claims data, we may not have included all appropriate patients. To reduce this limitation, the national registration system for rare incurable diseases was used, and variables were defined by combining diagnosis records and prescription records, a method verified in previous studies (10, 42, 43). Third, because serum GH or IGF-1 level was unavailable in claims data, GH or IGF-1 level could not be compared between patients with or without bronchiectasis. In addition, other respiratory diseases (asthma, chronic obstructive pulmonary disease, etc.) and underlying medical conditions (past history of tuberculosis or non-tuberculosis mycobacterial infections, smoking history, body mass index, etc.) that affect the occurrence of bronchiectasis could not be adjusted due to limitations of claims data and registry dataset we used. We therefore established a large control group by matching 1:5, thinking that we could correct for this confounding factor by including a very large number of subjects. Fourth, since this study was not prospective, it is not possible to determine a causal relationship between acromegaly and bronchiectasis. However, to reduce reverse causality, subjects diagnosed before the study period were excluded. Acromegaly is a disease that is difficult to study on a large scale due to its rarity. Although these limitations exist, in this study, we presented the results of a large-scale, long-term follow-up data on rare diseases using a well-validated cohort and real-world claims data managed by the government. Therefore, it is considered valuable to suggest future research based on this study.

In conclusion, this cohort study of patients with acromegaly showed higher incidence of bronchiectasis in patients with acromegaly compared with controls. This is the first large-scale study to examine the relationship between growth hormone excess state and bronchiectasis in a human disease model, and is significant in that it can be used as a basis for various future studies. Further studies are needed to investigate the causal relationship and pathophysiology of acromegaly on the development of bronchiectasis. Additionally, during the management of patients with acromegaly, attention should be paid to the development of bronchiectasis as well as other complications of acromegaly.

## Data Availability

The original contributions presented in the study are included in the article/**Supplementary Material**. Further inquiries can be directed to the corresponding author.
