# Supplementary material for: Incidence of bronchiectasis in patients with acromegaly: a cohort study
Source: Front Endocrinol (Lausanne). 2024 Aug 16;15:1362950. doi: 10.3389/fendo.2024.1362950 (PMC11361995; doi:10.3389/fendo.2024.1362950)
Supplement: Supplementary file 1 [file Table1.docx]

**Supplementary materials**

SUPPLEMENTARY TABLE 1 Subgroup analysis of frequency, incidence rate, and hazard ratio of bronchiectasis by baseline characteristics of subjects and the presence of interaction among variables

|  | No. patients | BE | Duration  (person-year) | Incidence rate  (per 1,000 person-year) | Hazard ratio  (95% confidence interval)* | P for interaction |
| --- | --- | --- | --- | --- | --- | --- |
| **Age: years** |  |  |  |  |  |  |
| < 65 |  |  |  |  |  |  |
| Control | 11395 | 211 | 103309 | 2.04 | 1 (ref.) | 0.3239 |
| Acromegaly | 2279 | 57 | 20288 | 2.81 | 1.377 (1.014–1.87) |  |
| ≥ 65 |  |  |  |  |  |  |
| Control | 1570 | 75 | 12320 | 6.09 | 1 (ref.) |  |
| Acromegaly | 314 | 25 | 2265 | 11.04 | 1.806 (1.14–2.863) |  |
| **Sex** |  |  |  |  |  |  |
| Male |  |  |  |  |  |  |
| Control | 5915 | 108 | 52693 | 2.05 | 1 (ref.) | 0.6513 |
| Acromegaly | 1183 | 33 | 10328 | 3.20 | 1.603 (1.077–2.384) |  |
| Female |  |  |  |  |  |  |
| Control | 7050 | 178 | 62936 | 2.83 | 1 (ref.) |  |
| Acromegaly | 1410 | 49 | 12224 | 4.01 | 1.427 (1.027–1.984) |  |
| **Income** |  |  |  |  |  |  |
| High |  |  |  |  |  |  |
| Control | 10204 | 215 | 91368 | 2.35 | 1 (ref.) | 0.3650 |
| Acromegaly | 2016 | 65 | 17685 | 3.68 | 1.591 (1.191–2.127) |  |
| Low 25% |  |  |  |  |  |  |
| Control | 2761 | 71 | 24261 | 2.93 | 1 (ref.) |  |
| Acromegaly | 577 | 17 | 4868 | 3.49 | 1.207 (0.706–2.065) |  |
| **Place** |  |  |  |  |  |  |
| Urban |  |  |  |  |  |  |
| Control | 6159 | 153 | 55462 | 2.76 | 1 (ref.) | 0.9293 |
| Acromegaly | 1230 | 45 | 10893 | 4.13 | 1.509 (1.071–2.126) |  |
| Rural |  |  |  |  |  |  |
| Control | 6806 | 133 | 60167 | 2.21 | 1 (ref.) |  |
| Acromegaly | 1363 | 37 | 11660 | 3.17 | 1.476 (1.014–2.148) |  |
| **Diabetes** |  |  |  |  |  |  |
| (-) |  |  |  |  |  |  |
| Control | 12190 | 253 | 109507 | 2.31 | 1 (ref.) | 0.6288 |
| Acromegaly | 1860 | 53 | 16627 | 3.19 | 1.552 (1.15–2.096) |  |
| (+) |  |  |  |  |  |  |
| Control | 775 | 33 | 6122 | 5.39 | 1 (ref.) |  |
| Acromegaly | 733 | 29 | 5925 | 4.89 | 1.345 (0.815–2.219) |  |
| **Hypertension** |  |  |  |  |  |  |
| (-) |  |  |  |  |  |  |
| Control | 10699 | 204 | 96484 | 2.11 | 1 (ref.) | 0.7444 |
| Acromegaly | 1592 | 39 | 14323 | 2.72 | 1.438 (1.014–2.04) |  |
| (+) |  |  |  |  |  |  |
| Control | 2266 | 82 | 19145 | 4.28 | 1 (ref.) |  |
| Acromegaly | 1001 | 43 | 8230 | 5.23 | 1.564 (1.073–2.279) |  |
| **Dyslipidemia** |  |  |  |  |  |  |
| (-) |  |  |  |  |  |  |
| Control | 11787 | 238 | 106589 | 2.23 | 1 (ref.) | 0.3277 |
| Acromegaly | 2150 | 65 | 19151 | 3.39 | 1.593 (1.197–2.121) |  |
| (+) |  |  |  |  |  |  |
| Control | 1178 | 48 | 9040 | 5.31 | 1 (ref.) |  |
| Acromegaly | 443 | 17 | 3401 | 5.00 | 1.17 (0.668–2.05) |  |

Abbreviation: BE: bronchiectasis.

^*^ Hazard ratios are adjusted for age, sex, household income, place, type 2 diabetes, hypertension, and dyslipidemia (Model 2)
